# Modulation of peroxisome proliferator activated receptor genes and gamma glutamyl transferase in rats by high intensity interval training and livergol

**DOI:** 10.1038/s41598-025-31956-5

**Published:** 2025-12-17

**Authors:** Farah Nameni, Akram Kasiri

**Affiliations:** Department of Physiology Exercise, VaP.C., Islamic Azad University, Varamin, Iran

**Keywords:** Livergol, High-intensity interval training, Proliferator-Activated receptor, Gamma-Glutamyl transferase, Genes, Diseases, Gastroenterology, Physiology

## Abstract

Obesity is a global health concern affecting multiple organs and is influenced by genetics, diet, and physical activity. Peroxisome proliferator-activated receptor gamma (PPARγ) regulates lipid and glucose metabolism, while gamma-glutamyl transferase (GGT) is a marker of liver function.The combined effect of high-intensity interval training (HIIT) and livergol, a liver-protective supplement, on these markers in obesity remains unclear. The primary aim of this study was to specifically examine the effects of 8 weeks of HIIT and livergolsupplementation, administered individually and in combination, on the gene expression levels of PPARγ and the enzymatic activity of GGT in the liver and adipose tissues of obese male Wistar rats. In this experimental study, forty male Wistar rats (8 weeks old, weighing 246 ± 15 g) were fed a high-fat diet to induce obesity, then randomly divided into four groups (*n* = 10): control, HIIT, livergol, and HIIT + livergol. The interventions lasted for 8 weeks. The study setting was laboratory-based. Adipose tissue and liver samples were collected one day after the last session in a fasting state and analyzed in the reference laboratory. The primary outcome measures were PPARγ gene expression (RT-PCR) in adipose tissues and GGT enzymatic activity (biochemical assay) in liver samples. Data were analyzed using Shapiro-Wilk, Levene, two-way ANOVA, and Bonferroni post hoc (*P* ≤ 0.05) tests. Results are presented as mean ± standard deviation with 95% confidence interval (CI). Among 40 obese male Wistar rats, HIIT combined with Livergolsupplementation markedly improved metabolic health by substantially upregulating PPARγ expression in the HIIT and HIIT + Livergolgroups (*P* < 0.05) while reducing GGT activity compared to controls (where GGT was significantly higher(*P* < 0.05). PPARγ expression increased by 317% in the HIIT group, 267% in the livergolgroup, and 435% in the HIIT + livergolgroup compared with controls. GGT activity decreased by 27.4%, 32.3%, and 23.9% in HIIT, livergol, and HIIT + livergolgroups, respectively, compared with the control group. Enhanced expression of PPARγ likely drives improved adipocyte differentiation, fatty acid oxidation, and insulin signaling in adipose and liver tissues, thereby attenuating dyslipidemia and hyperglycemia; these effects are amplified in the combined group. Simultaneously, the observed reductions in GGT activity indicate diminished hepatic oxidative stress and inflammation. Livergol’s silymarin content appears to potentiate this hepatoprotection by scavenging reactive oxygen species, and High-Intensity Interval Training (HIIT) emerges as a promising adjunctive strategy for obesity management.

## Introduction

Overweight and obesity represent urgent global health challenges that exert profound epigenetic influences and contribute to the development of liver disorders. These conditions arise from the complex interplay between genetic predisposition, sedentary behavior, dietary patterns, and physical inactivity. Multifaceted interventions, including nutritional modification, weight reduction, and structured exercise programs, have demonstrated significant therapeutic potential^1^.From a molecular perspective, high-intensity interval training (HIIT) in animal models activates fatty acid oxidation pathways and suppresses lipid synthesis by upregulating peroxisome proliferator-activated receptors (PPARs). This training modality may modulate PPAR gene expression and γ-glutamyltransferase(GGT) levels. GGT is a sensitive biomarker of oxidative stress and liver dysfunction, and evidence indicates that PPAR activation induced by regular exercise can reduce hepatic fat accumulation and subsequently decrease GGT levels^2^.

HIIT, characterized by alternating intervals of intense activity and recovery, imposes substantial physiological demands that trigger adaptive metabolic and molecular responses^3^. Such training has been shown to modulate hepatic lipid deposition and alter gene expression profiles. Parallel to exercise-based strategies, herbal interventions—with generally favorable safety profiles—have gained increasing attention. Recent evidence indicates that HIIT induces systemic adaptations beyond energy metabolism, improving bone remodeling, lipid profile, and physical function even in populations with chronic disease, such as individuals with multiple sclerosis These findings highlight the ability of HIIT to activate multisystem anabolic and anti-inflammatory pathways^4^.This reinforces the concept that HIIT exerts broad effects on lipid metabolism and oxidative stress.

HIIT is associated with robust signals of metabolic stress, inflammation, and acute muscle damage, which may indirectly modulate PPARγ and GGT.HIIT can elicit an intense metabolic response characterized by increased acute muscle damage and temporary metabolic dysfunctions, as reported in weight-category athletes undergoing rapid weight loss (Acute muscle damage as a metabolic response to rapid weight loss in wrestlers^5^. Although the physiological contexts differ (dehydration vs. exercise), both scenarios demonstrate how high-intensity stressors and High-intensity interval training improves bone remodeling, lipid profile, and physical function in multiple sclerosis patients.

Livergol, a formulation enriched with silymarin, exemplifies this approach through its potent antioxidant properties and its ability to support hepatocyte regeneration. In animal studies, HIIT has been shown to upregulate PPAR-α and PPAR-γ expression. For instance, mice fed a high-fat diet and subjected to HIIT exhibited a significant increase in PPAR-α expression; a change associated with enhanced fatty acid oxidation and reduced hepatic fat accumulation^6^.

For HIIT, the primary concerns include the elevated risk of musculoskeletal injury in sedentary, overweight individuals^7^.

Regarding livergol, while generally safe, it can cause mild gastrointestinal side effects, but its most significant disadvantage is the potential for critical drug interactions: Silymarin can modulate Cytochrome P450 enzymes in the liver, thereby altering the metabolism and blood concentration of other vital medications often taken by obese patients, which can compromise their efficacy or increase toxicity^8^.

Evidence also suggests synergistic effects between exercise and silymarin supplementation. In healthy men, combined silymarin intake and aerobic exercise decreased serum interleukin-6 (IL-6) and C-reactive protein (CRP), indicating enhanced anti-inflammatory potential^9^. Although conventional pharmacological treatments for liver disorders—such as interferons and corticosteroids—are mechanistically justified, their clinical use is often limited by inconsistent outcomes and adverse effects. Consequently, plant-derived compounds have emerged as promising alternatives to synthetic agents. Sobolev et al. (2022),[10] for example, reported that four weeks of silymarin supplementation significantly reduced serum aspartate aminotransferase (AST) and alanine aminotransferase (ALT) levels in patients with liver impairment. Findings from preclinical rodent models further confirm the hepatoprotective effects of silymarin against xenobiotic-induced and drug-induced hepatic injury^10^.

Parallel research has elucidated the transcriptional regulation and pleiotropic functions of PPARγ, a nuclear receptor isoform essential for lipid homeostasis^10^. Agonist-mediated activation of PPARγ has been examined extensively in mouse models, demonstrating isoform-specific differences in chromosomal localization, ligand-binding kinetics, target-gene activation, and metabolic regulation. Collectively, PPAR isoforms modulate key pathways related to inflammation, adipogenesis, and insulin sensitivity, and dysregulation of these pathways is strongly linked to obesity and type 2diabetes. Although exercise-based interventions have been shown to reprogram hepatic transcriptomes, their effects on circulating lipid profiles and aminotransferase levels remain inconsistent across studies^11^.

The primary aim of the present study is to evaluate the therapeutic potential of a combined intervention in a mouse model to improve liver function and mitigate metabolic disturbances. Specifically, it is hypothesized that HIIT stimulates PPAR gene expression, while livergol through its potent antioxidant and hepatoprotective properties—enhances hepatic regeneration. This combined approach allows the investigation of potential synergistic effects on reducing inflammation, improving metabolic markers, and restoring hepatocyte integrity. Clinically, such findings may contribute to the development of non-pharmacological therapeutic strategies that highlight the importance of interval training and natural supplements in preventing disease progression, reducing dependence on pharmacological agents, and enhancing quality of life—an approach particularly relevant in populations with a high prevalence of obesity and metabolic syndrome^12^.

**Fig. 1:**
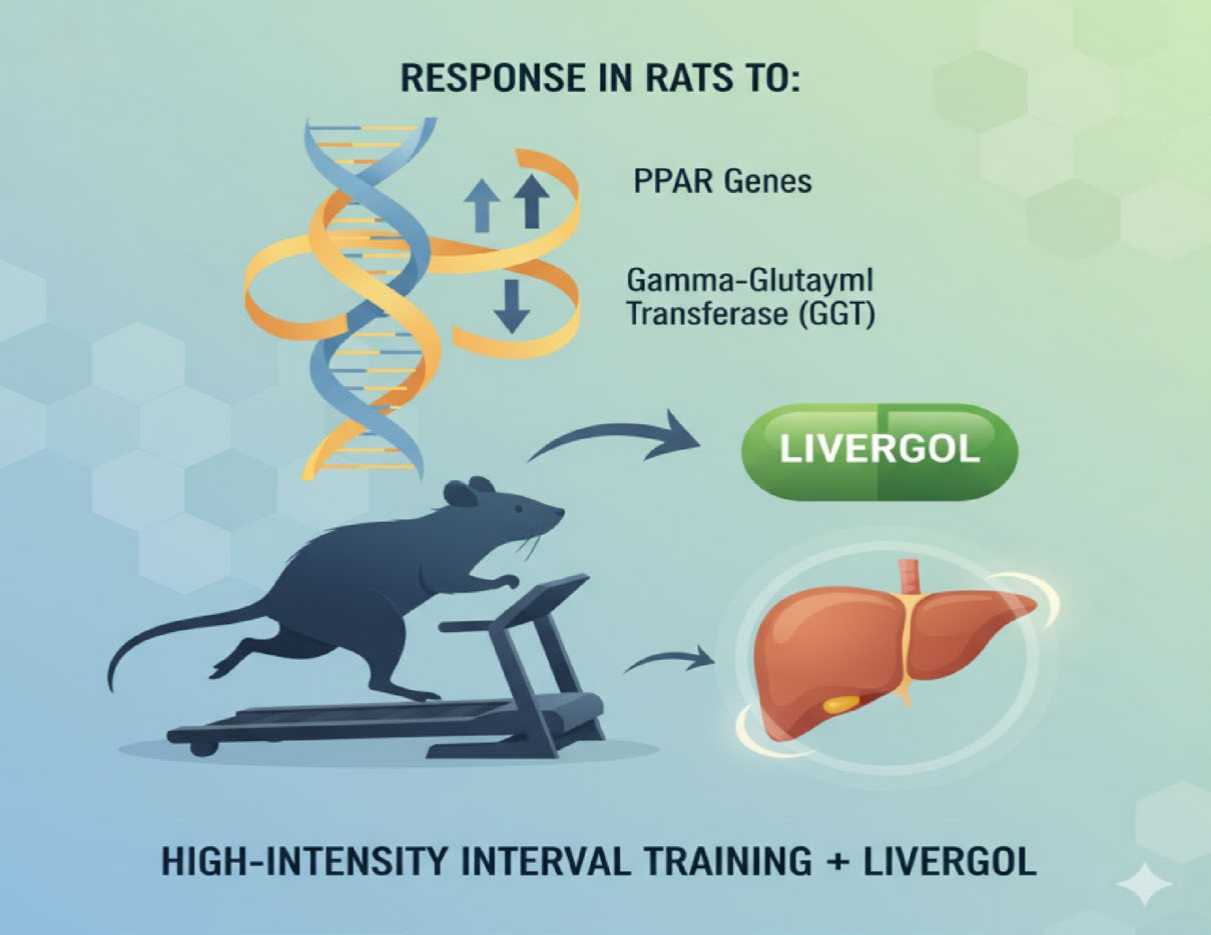
Demonstrating the relationship between high-intensity interval training and liver gel with peroxisome proliferator-activated receptor gene and gamma-glutamyl transferase in rats.

Despite the well-documented metabolic benefits of HIIT and the established hepatoprotective effects of livergol, limited research has explored their combined impact on molecular regulators of hepatic metabolism—specifically PPARs and GGT, a key enzyme involved in oxidative stress responses and liver function. Elucidating the molecular interactions between these interventions may uncover synergistic mechanisms that attenuate hepatic inflammation and promote liver regeneration. Such mechanistic insights are essential for designing effective non-pharmacological approaches to manage liver dysfunction and obesity-related metabolic disorders, particularly among high-risk groups. Accordingly, the central question of the present study is as follows: Does the combined administration of livergol and high-intensity interval training modulate PPARγ gene expression and GGT enzyme activity in obese male rats? Fig. 1.

## Research methodology

The primary hypothesis of this study was that the combined intervention of HIIT and livergol would modulate PPAR gene expression, reduce GGT activity, and exert synergistic effects on inflammation reduction and liver regeneration. This research employed a fundamental–applied experimental design with a post-test framework and four study groups. The research subjects consisted of adult male Wistar rats, selected as the target population due to their physiological relevance in metabolic and hepatic studies. A total of 50 rats (initial weight: 246 ± 15 g) were obtained from the Laboratory Animal Breeding and Housing Center of the Pasteur Institute of Iran and transferred to the university’s Animal Science Research Laboratory.

Upon transfer, animals underwent a one-week acclimatization period to minimize stress associated with environmental change. The rats were housed in pairs in transparent polycarbonate cages (30 × 15 × 15 cm; Razi Rad Manufacturing Company, Iran) under standard laboratory conditions, including a controlled ambient temperature of 22 ± 3 °C, a 12:12-hour light–dark cycle, and ad libitum access to non-standardized pelleted feed and drinking water.

All animal procedures strictly adhered to international ethical regulations, including the ARRIVE guidelines, the UK Animals (Scientific Procedures) Act (1986), the European Union Directive 2010/63/EU for the protection of animals used for scientific purposes, and the National Institutes of Health Guide for the Care and Use of Laboratory Animals^13^. Ethical approval for this study was obtained from the Institutional Review Board of the Faculty of Medicine, Islamic Azad University, Varamin-Pishva Branch (Approval ID: IR.IAU.VARAMIN.REC.1399.006).

### Randomization and sample size determination

Animals were randomly assigned using block randomization into four equal groups: Control, HIIT, livergol, and HIIT + livergol. Randomization was stratified by body weight (≤ 250 g and > 250 g), and within each stratum, allocation sequences were generated using the RAND function in Microsoft Excel and implemented in blocks of four. Allocation codes were sealed in opaque, numbered envelopes and remained confidential until the time of assignment. The investigator responsible for molecular and biochemical analyses was blinded to group allocation. The sample size was determined using the formula for comparing two independent means:

$$n=2(Z1-\alpha/2+Z1-\beta)2\frac{\Delta2}{\sigma2}$$

where α = 0.05 and statistical power (1 − β) = 0.80. Based on pilot data (Δ = 1.0, σ = 0.8), the required sample size was estimated to be *n* = 9 per group. To account for a potential 15% dropout rate, the final number of animals was increased to 10 per group.

### Experimental design and intervention protocol

To induce weight gain and create a high-calorie diet model in rats, a high-calorie diet enriched with fat and simple carbohydrates was used. The diet was formulated to contain approximately 4.5–5.2 kcal/g, which is significantly higher than the ~ 3 kcal/g of the standard chow (Table 1)^14^.

**Table 1:** Composition of the experimental diet (per 100 g) and macronutrient energy contribution.

Component	Amount (g/100 g)	Energy contribution (%)
Fat	25	40–45
Carbohydrates	45	35–40
Protein	20	15–20
Fiber (Cellulose)	5	—
Vitamin & Mineral mix	5	—

This high-calorie diet led to a significant increase in body weight. Male rats exhibited weight gain from 246 g to approximately 330–350 g following six weeks of dietary intervention. All animals were weighed twice weekly, and weekly growth curves were generated for each experimental group. Daily food and water intake were recorded to determine the average energy consumption per cage. At the end of the experimental protocol, the Lee Index was calculated as an indicator of adiposity and metabolic status using the following formula:

Lee Index = [Body Weight^(1/3) (g)]/[Naso–Anal Length (cm)]

This diet induced general weight gain and metabolic alterations, including increased triglycerides, hepatic steatosis, and insulin resistance. Resting heart rate was measured using a tail-cuff plethysmography system. Before recording, rats were placed in a quiet environment for several minutes to minimize stress and ensure accurate measurements. The behavioral status of each rat was monitored daily and assessed based on parameters such as locomotor activity, self-grooming, responsiveness to environmental stimuli, and signs of stress or lethargy. Animals were evaluated using a six-point scale, where 6 indicated a highly active and responsive state, and 1 represented lethargy or severe stress.

The study included four groups of ten rats each: Control, Exercise, livergol, and Exercise + livergol. All animals were acclimated to the laboratory environment and familiarized with the main training protocol before the intervention. The Exercise group underwent an eight-week high-intensity interval training (HIIT) program, whereas the livergol group received livergol supplementation for the same duration. The Exercise + livergol group received the supplement concurrently with the eight-week HIIT regimen (Table 2).

**Table 2:** High-intensity interval training program^15^.

Week	Odd days (intensity)*	Even days (high intensity)	Rest between bouts	Incline *(°)	Sessions/week	Warm-up/cool-down	Progression
1	2 × 3 min at 40 m/min	3–5 × 30 s at 54 m/min	60 s active rest at 16 m/min	≥ 5٪	5	5 min at 16 m/min (before & after)	Start phase
2–3	3 × 3 min at 40 m/min	5–7 × 30 s at 54 m/min	60 s active rest at 16 m/min	≥ 5٪	5	5 min at 16 m/min (before & after)	Gradual increase
4–5	4–5 × 3 min at 40 m/min	7–9 × 30 s at 54 m/min	60 s active rest at 16 m/min	≥ 5٪	5	5 min at 16 m/min (before & after)	Increased load
6–7	5–6 × 3 min at 40 m/min	9–11 × 30 s at 54 m/min	60 s active rest at 16 m/min	≥ 5٪	5	5 min at 16 m/min (before & after)	Near-maximum load
8	6 × 3 min at 40 m/min	15 × 30 s at 54 m/min	60 s active rest at 16 m/min	≥ 5٪	5	5 min at 16 m/min (before & after)	Peak phase

*: 70–85% of VO₂max or a speed that induces a serum lactate increase of approximately 6–10 mmol/L.

Livergol tablets were procured from Gol Darou Herbal Pharmaceutical Company (Isfahan, Iran) under a valid health license issued by the Iranian Food and Drug Administration (Batch No.LG-2401, Certificate of Analysis COA-2024-117). Each tablet contains 140 mg of standardized Silybum marianum extract, corresponding to approximately 70–80% silymarin, with silibinin as the principal active flavonolignan. The content and purity of silymarin were verified using high-performance liquid chromatography (HPLC). For analysis, extract samples were dissolved in a methanol–water solvent and injected into a C18 column of an HPLC system equipped with a UV detector set at 288 nm. Silymarin was identified and quantified by comparison with a pure standard, ensuring precise measurement of active flavonolignan concentrations and confirming the standardization of each tablet^16^.

For experimental administration, livergol tablets were finely powdered and freshly suspended daily in 0.5% carboxymethyl cellulose (CMC) solution to achieve the desired concentration. The solution was prepared at room temperature to ensure complete solubility and prevent precipitation. Fresh preparations were used for each administration. Livergol was delivered orally via gavage at a dose of 300 mg/kg body weight once daily between 8:00 and 9:00 am. This dosage was selected based on previous animal studies demonstrating hepatoprotective and antioxidant effects without significant toxicity and corresponds approximately to the human-equivalent dose when scaled for body surface area in rats^17^.

After gavage, animals were monitored for 3–5 min to ensure complete ingestion, and only those that had fully swallowed the solution were returned to their home cages. This procedure was performed consistently to ensure accurate dosing and reliable experimental outcomes^18^. The Control and Exercise + Placebo groups received maltodextrin in an identical preparation, ensuring that all four groups were equally exposed to the physiological effects of gavage.

A motorized treadmill (model A1400Y10, Pishro Andisheh Sanat Company, Iran) was used for the exercise protocol, with the slope maintained at zero throughout all sessions^19^. To reduce biological variability and enhance internal validity, the study was limited to male rats. The Control group did not receive any supplements but participated in the HIIT program.

Twenty-four hours after the final training session, rats were anesthetized via intraperitoneal injection of ketamine (90 mg/kg) combined with xylazine (10 mg/kg) to minimize potential confounding effects of exercise or other variables. Euthanasia was then performed with a lethal intraperitoneal dose of sodium pentobarbital (200 mg/kg). Upon confirmation of deep anesthesia, a midline laparotomy was conducted to carefully harvest epididymal adipose tissue for PPARγ analysis and medial hepatic lobes for GGT assessment. Collected tissues were separated from surrounding subcutaneous fat, rinsed with physiological saline (0.9%NaCl), and rapidly frozen in liquid nitrogen before storage at − 80 °C for subsequent molecular and enzymatic analyses. All experimental procedures complied with institutional guidelines for the care and use of laboratory animals and were approved by the Institutional Animal Care and Use Committee (IACUC).

### PPARγ gene expression study using the RT-PCR technique

Based on the main hypotheses of the study, the combined intervention of HIIT and the livergol primer for PPARγ gene expression was designed (Table 3).

**Table 3:** Primer information for examining PPARγ gene expression.

Target gene	Product size (bp)	Tm (°C)	Primer sequence (5′→3′)	Direction	Source/References
PPARγ	150	60	AGGCCGAGAAGGAGAAGCTG	Forward	Designed using NCBI Primer-BLAST
			TGGCCACCTCTTTGCTCTA	Reverse	Designed using NCBI Primer-BLAST
β-actin	120	60	AGAGGGAAATCGTGCGTGAC	Forward	Designed using NCBI Primer-BLAST
			CAATAGTGATGACCTGGCCGT	Reverse	Designed using NCBI Primer-BLAST
GAPDH	123	62.6	AGGTCGGTGTGAACGGATTTG	Forward	PrimerBank ID: 6679937a1
GAPDH	123	60.2	TGTAGACCATGTAGTTGAGGTCA	Reverse	PrimerBank ID: 6679937a1

RNA sequencing (RNA-seq) provided a comprehensive analysis of the entire transcriptome. This approach enabled the identification of new pathways and regulatory networks related to metabolism and oxidative stress. Primer design criteria included specificity, melting temperature of approximately 60 °C, absence of significant secondary structures, and amplicon size of 100–200 bp. For RNA extraction and cDNA synthesis, total RNA was isolated from tissues using a Qiagen RNA extraction kit (Qiagen, Germany) according to the manufacturer’s protocol. RNA quantity and purity were assessed using a Nanodrop spectrophotometer (Thermo Scientific, USA), and only samples with an A260/A280 ratio between 1.8 and 2.0 were used for cDNA synthesis. Reverse transcription was performed with the Qiagen cDNA synthesis kit following the manufacturer’s instructions.

qPCR reactions were performed in triplicate using SYBR Green dye on a Real-Time PCR system (Applied Biosystems, USA). Thermal cycling conditions were: initial denaturation at 95 °C for 5 min, followed by 35 cycles of 95 °C for 30 s, 60 °C for 30 s, and 72 °C for 30 s, with a final extension at 72 °C for 5 min.

Validation and quality control:

Amplification efficiency for each primer pair was determined by generating standard curves from a 5-fold serial dilution of cDNA; efficiencies ranged between 90 and 105%.

Melt curve analysis was performed at the end of each run to confirm the specificity of amplification, ensuring a single peak per primer pair. Selected PCR products were further validated by agarose gel electrophoresis to confirm the expected product size. The stability of β-actin expression was confirmed across all experimental groups; additionally, a second housekeeping gene (GAPDH) was assessed to ensure normalization reliability. Relative gene expression was calculated using the ΔΔCt method. First, the difference in Ct values between the target and reference gene (ΔCt) was determined for each sample. Then, the ΔCt of the intervention groups was compared with the control group to obtain ΔΔCt. Fold change in gene expression was calculated as 2^-ΔΔCt and reported accordingly^20^.

### Investigation of GGT biochemical index

Following confirmation of profound anesthesia in the rats (injection error < 5%, per USP pharmacopeial standards), the abdominal cavity was accessed via midline laparotomy to enable meticulous hepatic excision. The liver was immediately irrigated with ice-cold phosphate-buffered saline (PBS; pH7.4) to remove residual erythrocytes and extraneous debris. A representative hepatic aliquot (0.2–0.5 g) was homogenized in a 1:9 (w/v) ratio with chilled 0.1 M Tris-HCl buffer (pH7.4) using a Teflon-glass pestle-and-mortar under cryogenic conditions to prevent denaturation of thermolabile enzymatic components. The homogenate was then ultracentrifuged at 10,000×g for 15 min at 4 °C, yielding a clarified supernatant for enzymatic profiling.

GGT catalytic activity was quantified spectrophotometrically using the classical γ-glutamyl transpeptidation method. The reaction mixture contained 1.0 mM γ-glutamyl-p-nitroanilide (γ-glutamyl donor) and 40 mM glycylglycine (acceptor substrate) in 0.1 M Tris-HCl buffer (pH 8.2). The reaction was initiated by adding 0.1 mL of the supernatant to 0.9 mL of the preincubated substrate mixture at 37 °C. Hydrolysis and transfer of the γ-glutamyl residue resulted in liberation of p-nitroaniline, which was quantified by measuring absorbance at 405 nm. Measurements were performed using the Pars Azmoun GGT enzyme kit (Iran; containing 1.0 mM γ-glutamyl-p-nitroanilide and 40 mM glycylglycine in Tris-HCl buffer, pH 8.2; sensitivity: 1 µmol/min; accuracy: ±5%) in conjunction with an Iranian Nano spectrophotometer (resolution: 0.1–1 nm; absorbance accuracy: ±0.002; error: <0.5%). Protein content of the homogenate was determined using the DNAbiotech Bradford assay kit (Iran; sensitivity: 3 µg/mL; accuracy: ±2–5%; linearity:3–1000 µg/mL) at 595 nm.

Enzyme-specific activity was expressed in units per milligram of protein (U/mg protein), with one unit (U) defined as the amount of enzyme catalyzing the release of 1 µmol of p-nitroaniline per minute under the specified assay conditions. Normalization was based on the Bradford Coomassie Brilliant Blue G-250 dye-binding method. The assay demonstrated good reproducibility, with intra-assay coefficients of variation (CVs) ≤ 10% and inter-assay CVs ≤ 15%, ensuring the accuracy and consistency of the measurements. Samples were stored at − 80 °C to preserve enzyme integrity and prevent the effects of multiple freeze–thaw cycles.

### Statistical methods and data analysis

Descriptive statistics, including mean values, standard deviations, and tabular representations, were used to summarize the data. The normality of data distribution was assessed using the Shapiro-Wilk test, and homogeneity of variance was evaluated with Levene’s test. Comparisons of mean changes between groups were performed using two-way analysis of variance (ANOVA). When significant differences were detected, the Bonferroni post hoc test was applied. Statistical significance was set at *P* ≤ 0.05. All analyses were conducted using SPSS version 22 and Microsoft Excel 2010.

The outcome measures of this study included PPAR-γ gene expression, assessed using Real-Time PCR—a method that, due to its high sensitivity, reproducibility, and use of stable reference genes, has a coefficient of variation of 1–3% and a repeatability greater than 0.98, as well as validity with an efficiency of 90–110% and a coefficient of determination of 0.99—and GGT enzyme activity, measured spectrophotometrically, with a CV of 3–7% and standard validity reflected by a coefficient of determination greater than 0.97. The accuracy and reliability of these measurements were further enhanced by kit quality, temperature standardization, and spectrophotometer calibration. Body weight was also recorded using a sensitive digital scale with an error of less than 1%. Additionally, exercise performance indices, including maximal power and aerobic capacity, were evaluated with high reliability (ICC 0.85–0.95) and appropriate construct validity, particularly in HIIT protocols, which are both highly repeatable and considered valid indicators for assessing training adaptations.

## Results

### Growth and body composition indicators

Body weight, growth rate, and energy expenditure were assessed in all four experimental groups before the initiation of the protocol to ensure comparability. Baseline measurements confirmed that body weight, growth rate, and initial energy expenditure were equivalent across groups, ensuring that subsequent changes could be attributed to the interventions (HIIT and/or livergol) rather than pre-existing differences. During the intervention period, rats in all groups exhibited progressive increases in body weight; however, the rate of weight gain was significantly lower in the HIIT + livergol group compared to the control group (*p* < 0.05). The Lee index was also significantly reduced in the trained and supplemented groups, indicating improved body composition and decreased adiposity. No significant differences were observed in daily food or water intake among the groups, suggesting that the observed changes in body weight were primarily due to metabolic adaptations rather than alterations in energy intake (Table 4).

**Table 4:** Body weight, growth rate, and energy intake in different experimental groups.

Group	Initial body weight (g)	Final body weight (g)	Average growth rate (g/week)	Daily food intake (g/rat)	Daily energy intake (kcal/rat)
Control	246 ± 7	350 ± 20	10 ± 1	20 ± 0.5	60 ± 1.5
HIIT	250 ± 4	345 ± 15	9.5 ± 1	19 ± 0.4	57 ± 1.2
Livergol	246 ± 5	348 ± 10	9.8 ± 0.8	20 ± 0.5	59 ± 1.4
HIIT + Livergol	251 ± 2	340 ± 12	9 ± 0.8	19 ± 0.3	56 ± 1.0

Blood metabolic and biochemical indices (FBG, FINS, TC, TG, LDL-C, HDL-C, GGT, AST, ALT, and inflammatory markers) were measured and recorded for the groups (Table 5).

**Table 5:** Metabolic and biochemical blood parameters in different experimental groups.

Group	FBG (mg/dL)	FINS (µIU/mL)	TC (mg/dL)	TG (mg/dL)	LDL-C (mg/dL)	HDL-C (mg/dL)	GGT (U/L)	AST (U/L)	ALT (U/L)
Control	145 ± 8	22 ± 2	180 ± 12	150 ± 10	110 ± 8	38 ± 4	40 ± 4	70 ± 5	50 ± 4
HIIT	130 ± 7	18 ± 2	160 ± 10	130 ± 8	90 ± 6	45 ± 4	32 ± 3	60 ± 4	42 ± 3
Livergol	135 ± 7	19 ± 2	165 ± 10	135 ± 9	95 ± 7	42 ± 3	35 ± 3	65 ± 4	45 ± 3
HIIT + Livergol	125 ± 6	16 ± 1.5	155 ± 9	120 ± 7	85 ± 6	48 ± 3	28 ± 2	55 ± 3	40 ± 2

To investigate the effect of the high-intensity exercise program, heart rate, the animals’ time to fatigue and VO₂max were measured and recorded before, and after the protocol period (Table 6).

**Table 6:** Exercise performance in obese rats before and after 8 weeks.

Group	Baseline VO₂max (mL/kg/min)	VO₂max After 8 Weeks	Baseline Time to Exhaustion (min)	Time to Exhaustion After 8 Weeks (min)	Heart Rate Week 0 (bpm)	Heart Rate Week 8 (bpm)
Control	37 ± 2	38 ± 2	12 ± 1.5	12 ± 1.5	360 ± 15	362 ± 15
HIIT	38 ± 2	49 ± 3	12 ± 1.5	19 ± 1.8	358 ± 14	370 ± 12
Livergol	37.5 ± 2	40 ± 2	12 ± 1.5	13 ± 1.3	359 ± 15	365 ± 13
HIIT + Livergol	38 ± 2	51 ± 3	12 ± 1.5	21 ± 1.5	358 ± 14	372 ± 12

**Fig. 2:**
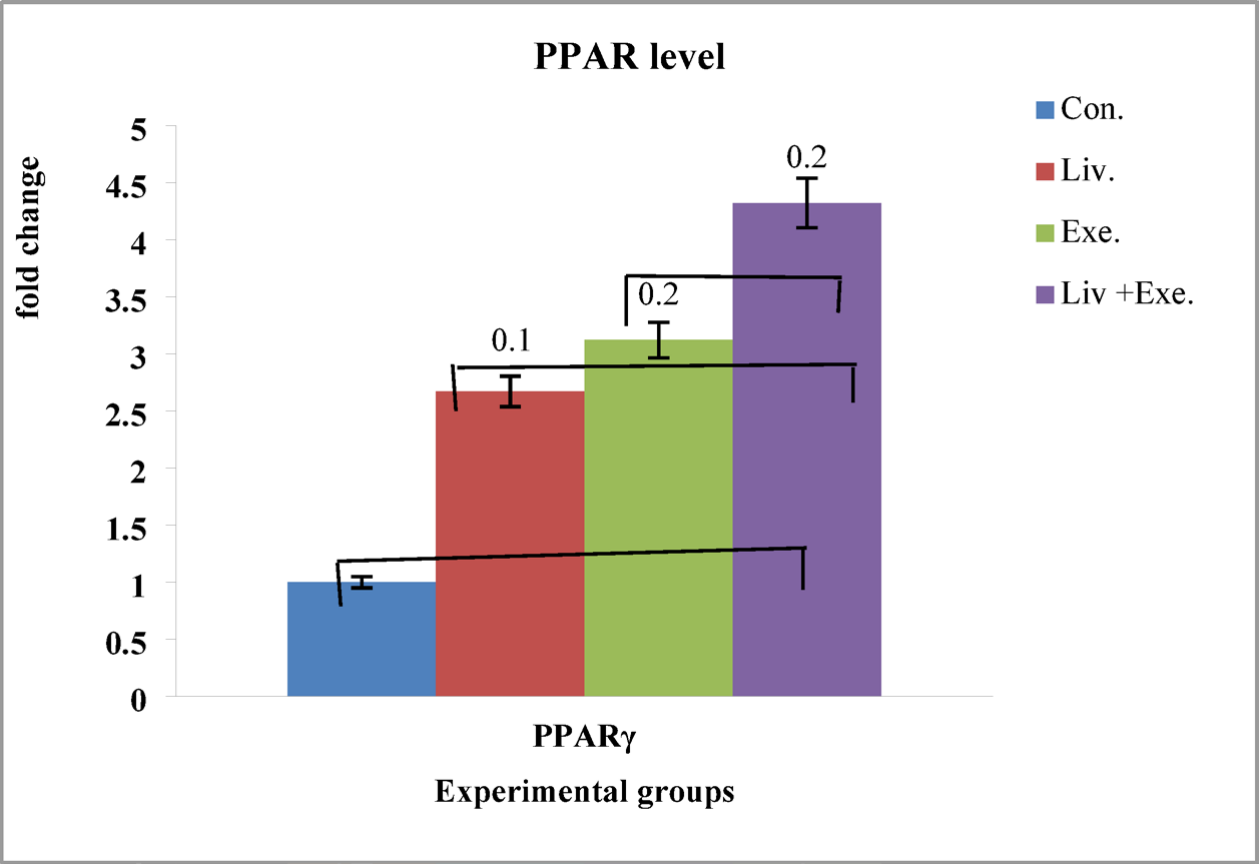
Changes in PPARγ in adipose tissue of the four studied groups. Effect size of intervention and magnitude of change groups for PPARγ. Exercise group vs. control group: 1.8. Liverpool vs. control: 1.7. Livergol + exercise vs. control: 2.9.

The changes in PPARγ gene expression between the four control and experimental groups were calculated and recorded (Fig. 2).

The results indicated that PPARγ gene expression significantly increased in response to exercise (F^1,24^ = 13.170, *p* = 0.001, η² =0.338), livergol intake (F^1,24^ = 8.271, *p* = 0.005, η²=0.298), and their interaction (F^1,24^ = 23.163, *p* = 0.018, η²=0.498). Post-hoc Bonferroni tests revealed significant differences among groups, with 95% confidence intervals (CI) for mean differences as follows: exercise + livergol vs. control: 2.452–2.748, exercise + livergol vs. livergol: 1.302–1.598, and exercise + livergol vs. exercise: 1.052–1.348. Post-hoc power for detecting the interaction effect was 0.92, confirming adequate sensitivity. The main effect of Training was also significant (F^1,24^ = 13.17, *p* = 0.001, η² =0.338, Cohen’s d = 1.8), with sedentary rats showing lower PPARγ expression (2.275 ± 0.175, 95% CI: 1.95–2.60) compared with trained rats (3.7 ± 0.175, 95% CI: 3.38–4.02), and post-hoc power of 0.89. Similarly, livergol administration had a significant main effect (F^1,24^ = 8.27, *p* = 0.005, η²=0.298, Cohen’s d = 1.7), with Control rats at 1.7 ± 0.1 (95% CI:1.55–1.85) and livergol -treated rats at 3.575 ± 0.175 (95% CI:3.25–3.90), with post-hoc power of 0.87(Table 7).

**Table 7:** Results of analysis of variance for examining PPARγ gene expression and effect size.

Source of variation	df	F-value	*p*-value	η²	Cohen’s d	Mean ± SD (per group)
Training × Livergol Interaction	1, 24	23.16	0.018	0.498	2.6	Control: 1.7 ± 0.1 Livergol: 2.85 ± 0.15 Exercise: 3.1 ± 0.15 Livergol + Exercise: 4.3 ± 0.2
Training	1, 24	13.17	0.001	0.338	1.8	Sedentary: 2.275 ± 0.175 Training: 3.7 ± 0.175
Livergol Administration	1, 24	8.27	0.005	0.298	1.7	Control: 1.7 ± 0.1 Livergol: 3.575 ± 0.175

**Fig. 3:**
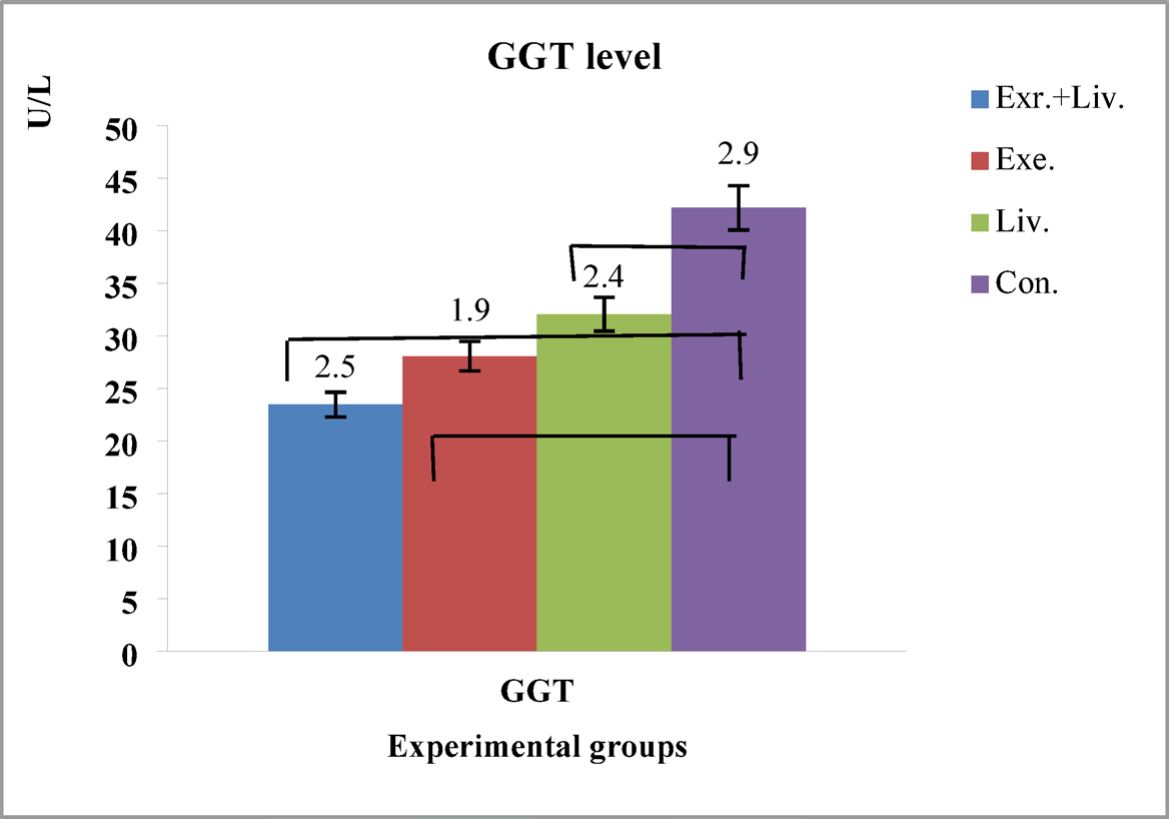
Changes in GGT in the four study groups in terms of international units. Effect size of intervention and magnitude of change groups for GGT. Exercise group vs. control group: 3.0. Liverpool vs. control: 2.8. Livergol + exercise vs. control: 3.2.

Changes in GGT concentration between the four control and experimental groups were calculated and recorded (Fig. 3).

Two-way ANOVA revealed significant main effects of Training (F^1,24^ = 29.543, *p* = 0.001, η² = 0.265, Cohen’s d = 3.0) and livergol administration (F^1,24^ = 34.245, *p* = 0.001, η²=0.210, Cohen’s d = 2.8) on GGT activity, as well as a significant Training × livergol interaction (F^1,24^ = 12.659, *p* = 0.001, η²=0.289, Cohen’s d = 3.2). These findings indicate that both HIIT and livergol independently modulated GGT activity, while their combined administration produced the most pronounced reduction. The mean ± SD values for each group were: control: 44.03 ± 0.21 U/L, livergol: 32.03 ± 0.2 U/L, exercise: 28.04 ± 0.12 U/L and exercise + livergol: 23.45 ± 0.1 U/L. Post-hoc Bonferroni comparisons revealed significant differences between all groups (*p* < 0.05). Two-sided 95% confidence intervals (CI) for mean differences were approximately: exercise + livergol vs. control:−20.73 to − 20.43 U/L, exercise + livergol vs. livergol: −8.48 to − 8.08 U/L, and exercise + livergol vs. exercise:−4.64 to − 4.24 U/L. Post-hoc power for detecting interaction and main effects ranged from 0.91 to 0.93, confirming adequate sensitivity. Overall, these results demonstrate that HIIT and livergol each significantly reduced GGT activity and their combination produced a synergistic effect. The inclusion of two-way ANOVA, Bonferroni-adjusted post-hoc tests, confidence intervals, effect sizes, and post-hoc power provides robust and reliable evidence for the impact of these interventions on oxidative stress and liver function (Table 8).

**Table 8:** Results of analysis of variance for GGT enzyme concentration and effect size (η²).

Source of variation	df	F (Value)	*p*-value	η²	Cohen’s d	Mean ± SD (per group)
Livorgol Administration	1, 24	34.245	0.001	0.210	2.8	Control: 44.03 ± 0.21 Livergol: 32.03 ± 0.2 Exercise: 28.04 ± 0.12 Livergol + Exercise: 23.45 ± 0.1
Training	1, 24	29.543	0.001	0.265	3.0	
Training×Livorgol Interaction	1, 24	12.659	0.001	0.289	3.2	

A study of pairwise Euclidean distances (absolute differences) was also performed (Tables 9 and 10).

**Table 9:** Pairwise Euclidean distances (absolute differences) in PPARγ variable gene expression across groups.

Groups	Control	Livergol	Exercise	Livergol + Exercise
Control	0.00	1.15	1.40	2.60
Livergol	1.15	0.00	0.25	1.45
Exercise	1.40	0.25	0.00	1.20
Livergol + Exercise	2.60	1.45	1.20	0.00

The greatest difference from the control group (d = 2.60) was observed in the combination group, indicating a synergistic increase in PPARγ expression, which plays a key role in lipid regulation and the reduction of inflammation. In contrast, the high similarity between the livergol and Exercise groups (d = 0.25) suggests comparable effects on PPARγ pathways. Notably, the combination treatment significantly altered the expression profile, consistent with the interaction effect observed in the two-way ANOVA (η² = 0.498).

**Table 10:** Pairwise Euclidean distances (absolute differences) in GGT variable gene expression across groups.

Groups	Control	Livergol	Exercise	Livergol + Exercise
Control	0.00	12.00	15.99	20.58
Livergol	12.00	0.00	3.99	8.58
Exercise	15.99	3.99	0.00	4.59
Livergol + Exercise	20.58	8.58	4.59	0.00

The greatest deviation from the control group (d = 20.58) was observed in the combination group, indicating a marked reduction in GGT a key marker of liver damage—and highlighting the protective effects of HIIT and livergol. The mean difference between the livergol and exercise groups (d = 3.99) suggests comparable effects on liver metabolism, whereas the combination treatment produced the most pronounced improvement, consistent with the interaction effect observed in ANOVA (η² =0.210).

Small effect sizes indicate that the intervention may have limited or inconsistent impact; the magnitude of the observed changes is minor, which may limit the practical or clinical significance of the findings. Wide confidence intervals reflect high uncertainty around the estimated effect, variability in response, and limited generalizability of the results. Together, these metrics suggest that, although statistical significance may be achieved, the actual effectiveness of the intervention may be modest and should be interpreted with caution.

**Fig. 4:**
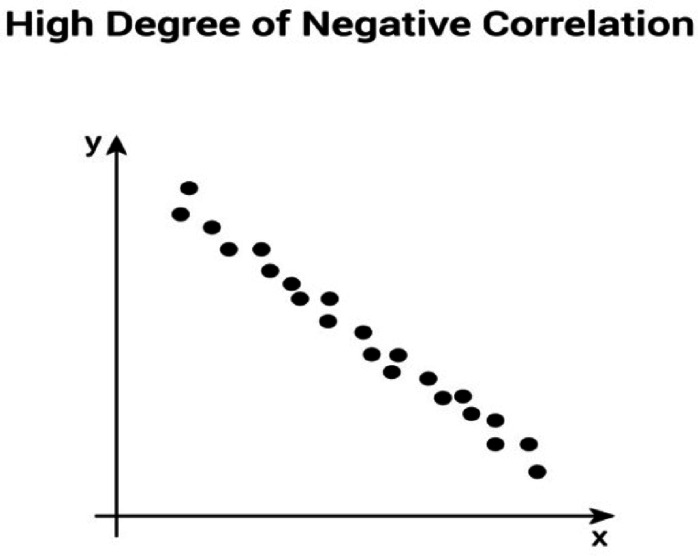
PPARγ uregulation correlates with decreased GGT levels following interventions.

The Pearson correlation coefficient between PPARγ fold change and GGT levels was approximately *r* = − 0.975, indicating a very strong and negative linear relationship between these two variables. This finding demonstrates that as PPARγ expression increases, GGT levels decrease, suggesting an inverse association between enhanced lipid regulation and reduced liver damage (Fig. 4).

**Fig. 5:**
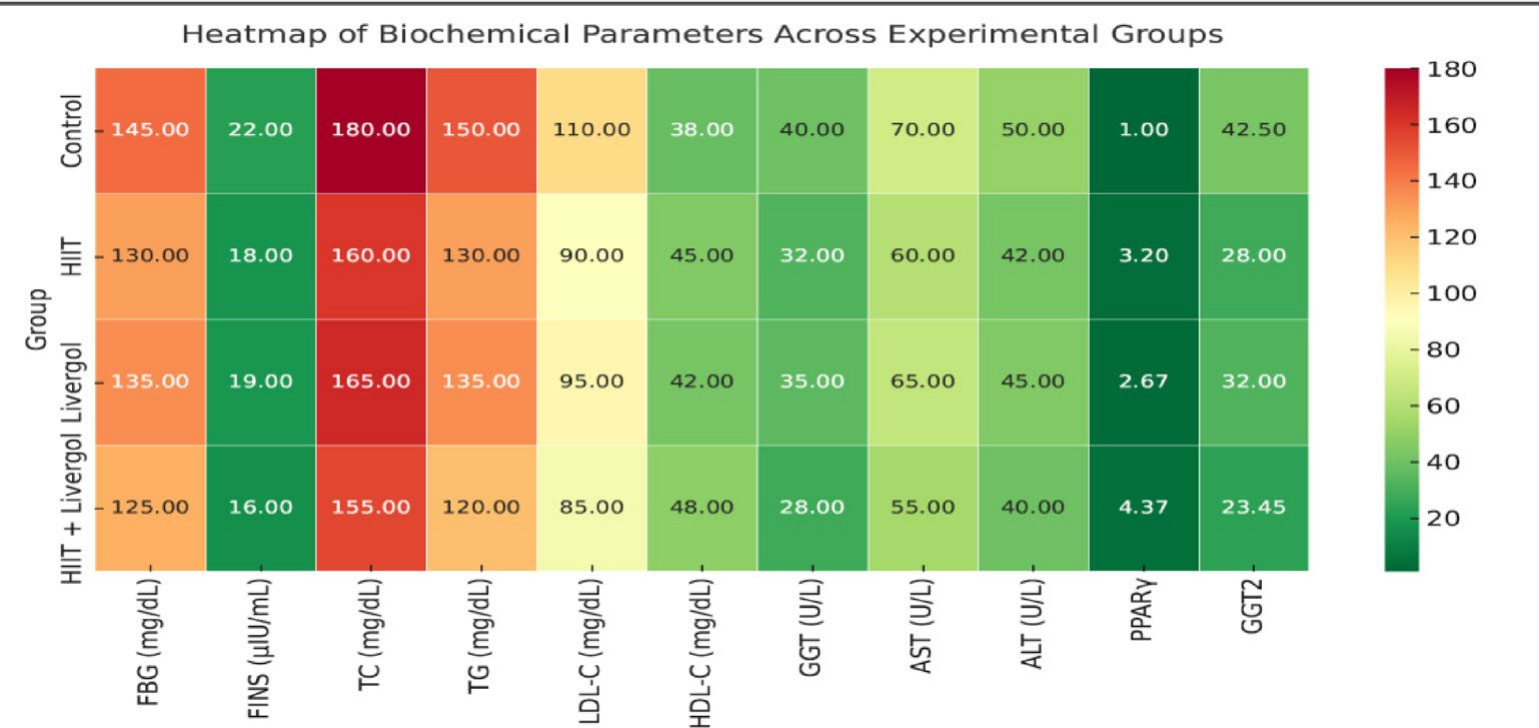
Correlation heatmap illustrating potential mechanistic links between PPARγ gene expression, GGT enzyme activity, and metabolic parameters.

A heat map illustrating the correlations among all measured variables in the study was generated, providing a visual representation of potential mechanistic links between PPARγ gene expression, GGT enzyme activity, and metabolic parameters (Fig. 5).

## Discussion

The results of this study demonstrated that high-intensity interval training and livergol supplementation increased PPARγ expression. PPARγ plays a central role in adipocyte differentiation, regulation of key lipid metabolism enzymes, hormone-sensitive lipase activity, and the differentiation of white and brown adipocytes. HIIT and livergol likely activated these mechanisms. Similarly, Kandel et al. (2024) reported that PPARγ regulates metabolism and modulates insulin sensitivity and glucose catabolism^21^. It is plausible that controlling plasma lipid levels and preventing their increase may further enhance PPARγ activity, a process improved by livergol supplementation^22^.

Exercise-induced oxidative stress may serve as a stimulus for upregulating PPARγ. Enhanced aerobic respiration and the utilization of lipids as fuel in skeletal muscle promote fatty acid mobilization from adipose tissue; further regulating PPARγ activity^23,24^. Livergol supplementation may also exert effects by reducing stress and enhancing metabolic pathways to promote PPARγ expression^25^.

Activation of PPARγ requires ligands, which include natural physiological molecules such as fatty acids and pharmacological agents to treat hyperglycemia and insulin resistance^26^. Accordingly, the combined effects of HIIT and livergol appear to robustly enhance PPARγ expression.

These findings contrast with those of Zhang et al. (2011) and Nazari et al. (2023)^27,28^, likely due to differences in exercise modality, intensity, duration, and volume. For example, Nazari et al. did not employ forced exercise, which may explain the lack of observed PPARγ upregulation. Zheng et al. (2025) investigated PPARα in relation to inflammation and atherosclerosis, but used a different exercise protocol and did not include livergol supplementation^29^. Po et al. (2022) studied the delta PPAR index in vascular pathophysiology without specific exercise or supplementation interventions, yielding results that are not directly comparable^30^.

Another notable outcome of this study was the reduction in hepatic GGT levels. HIIT, characterized by short and intense bouts of exercise, likely promoted fat catabolism and reduced metabolic stress in rats. Kamrul et al. (2024) reported that silymarin effectively decreased liver enzymes ALT and AST in patients with nonalcoholic fatty liver disease^31^. In contrast, Razzak (2024) found that environmental interventions alone had limited effects on enzyme levels. The GGT-lowering effects of HIIT and livergol may be mediated through enhanced β-oxidation of fatty acids, reduced triglyceride synthesis, and increased plasma HDL-C (high-density lipoprotein cholesterol) levels^32,33^.

Another potential mechanism underlying the reduction in metabolic stress involves the modulation of energy metabolism pathways by both livergol and high-intensity interval training. These interventions may inhibit acetyl-CoA carboxylase (ACC), limiting the conversion of acetyl-CoA to malonyl-CoA, thereby promoting fatty acid oxidation and suppressing lipid synthesis^34^.

In this study, livergol supplementation—containing flavonolignans (silybin, silyquercetin, isosilybin, silydianin), flavonoids, anti-inflammatory antioxidants, and immune modulators—reduced oxidative stress induced by interval training^16^. Gender differences may influence physiological and molecular responses, as well as patterns of gene regulation, antioxidant activity, and enzymatic adaptations, which differ from those observed in men. Estrogen protects mitochondrial function against oxidative stress, modulating PPARγ expression and GGT activity^35^. Progesterone may influence glucose utilization and glycogen storage dynamics. Women also rely more on lipid substrates and less on carbohydrate oxidation during submaximal exercise^36^. These sex-dependent metabolic strategies are partly determined by the hormonal environment and differential regulation of metabolic genes and signaling pathways^37^.

A key finding of the present study is the strong negative correlation observed between PPARγ expression and GGT activity (*r* = − 0.975), indicating a significant physiological response to high-intensity interval training and livergol supplementation. PPARγ acts as a master regulator of lipid metabolism and antioxidant gene expression, whereas GGT serves as a sensitive biomarker of oxidative stress and cellular damage. Therefore, increased PPARγ expression is associated with decreased GGT activity, reduced oxidative stress, and improved cellular function. This relationship is likely mediated through the AMPK–PPARγ axis, whereby activation of AMPK (adenosine monophosphate–activated protein kinase) induces PPARγ expression, enhances antioxidant defenses, and subsequently decreases GGT levels to maintain redox homeostasis. These findings suggest that exercise and liverwort supplementation can synergistically modulate metabolic and antioxidant pathways.

There is a substantial gap between diet-induced obesity models in rodents and the complexity of human metabolic diseases. Rats do not fully capture the diversity, temporal progression, or course of metabolic disorders in humans. High-fat diets in rats lack the macronutrient complexity of human diets, and genetic differences between strains contribute to variability in the onset and severity of metabolic symptoms. Therefore, observed changes in PPAR gene expression or enzyme activity, such as GGT, should be interpreted only as preliminary mechanistic insights. Species differences in metabolism, gene regulation, liver enzyme activity, and hormonal responses may influence how PPAR and gamma-glutamyl transferase signaling respond to exercise or pharmacological interventions. Moreover, the volume and intensity of exercise applied to rodents differ considerably from human exercise protocols. Consequently, further studies in human models are required to validate the translational relevance of these findings.

## Conclusion

In conclusion, the present study demonstrates that high-intensity interval training combined with livergol supplementation effectively enhances PPAR expression while reducing GGT activity. The upregulation of PPAR, a key regulator of lipid metabolism and antioxidant defenses, is associated with decreased oxidative stress and improved cellular function, as reflected by the concomitant reduction in GGT levels. These findings suggest a beneficial modulation of metabolic and redox homeostasis, highlighting the potential of exercise and nutritional interventions to synergistically improve metabolic health. Further research in human models is warranted to confirm the translational applicability of these results.

## Limitations

- Sample size: The number of animals studied may limit the statistical power for some sub-analyses.
- Intervention duration: The exercise protocol and livergol administration were short-term; long-term effects remain unknown.
- Pathway analysis: While the PPARγ–GGT axis was investigated, other relevant molecular pathways were not examined.
- Environmental and nutritional factors: Minor variations in diet or housing conditions may have influenced the outcomes.
- One limitation of the study is the real-time constraints faced by the researchers, which affected data collection, monitoring, and analysis. These time restrictions may have influenced the frequency and timing of measurements, the duration of interventions, or the depth of mechanistic investigations, potentially affecting the precision and completeness of the reported findings.

## Data Availability

Data is provided within the manuscript.

## Abbreviations

FBG: Fasting Blood Glucose
FINS: Fasting Insulin
TC: Total Cholesterol
TG: Triglycerides
LDL-C: Low-Density Lipoprotein Cholesterol
HDL-C: High-Density Lipoprotein Cholesterol
GGT: Gamma-Glutamyl Transferase
AST: Aspartate Aminotransferase
ALT: Alanine Aminotransferase
Inflammatory marker: TNF-α, IL-6, CRP
HIG2: hypoxia-inducible gene 2
AMPK: AMP-activated protein kinase
CPT1: Carnitine Palmitoyltransferase 1
ATGL: Adipose triglyceride lipase
Nrf2: Nuclear factor erythroid 2-related factor 2
ARE: Antioxidant Response Elements
